# Chemical Analysis of the Herbal Medicine *Salviae miltiorrhizae* Radix et Rhizoma (Danshen)

**DOI:** 10.3390/molecules21010051

**Published:** 2016-01-05

**Authors:** Hanqing Pang, Liang Wu, Yuping Tang, Guisheng Zhou, Cheng Qu, Jin-ao Duan

**Affiliations:** 1 Jiangsu Collaborative Innovation Center of Chinese Medicinal Resources Industrialization, Nanjing University of Chinese Medicine, Nanjing 210023, China; hanqingpang@126.com (H.P.); qucheng9527@163.com (C.Q.); dja@njucm.edu.cn (J.D.); 2 National and Local Collaborative Engineering Center of Chinese Medicinal Resources Industrialization and Formulae Innovative Medicine, Nanjing University of Chinese Medicine, Nanjing 210023, China; wuliang15@sohu.com; 3 Jiangsu Key Laboratory for High Technology Research of TCM Formulae, Nanjing University of Chinese Medicine, Nanjing 210023, China; zhouguisheng1@126.com

**Keywords:** Danshen, chemical analysis, quality control, polysaccharides

## Abstract

Radix *Salviae miltiorrhizae* et Rhizoma, known as Danshen in China, is one of the most popular traditional Chinese medicines. Recently, there has been increasing scientific attention on Danshen for its remarkable bioactivities, such as promoting blood circulation, removing blood stasis, and clearing away heat. This review summarized the advances in chemical analysis of Danshen and its preparations since 2009. Representative established methods were reviewed, including spectroscopy, thin layer chromatography, gas chromatography, liquid chromatography (LC), liquid chromatography-mass spectrometry (LC-MS), capillary electrophoresis, electrochemistry, and bioanalysis. Especially the analysis of polysaccharides in Danshen was discussed for the first time. Some proposals were also put forward to benefit quality control of Danshen.

## 1. Introduction

**Figure 1:**
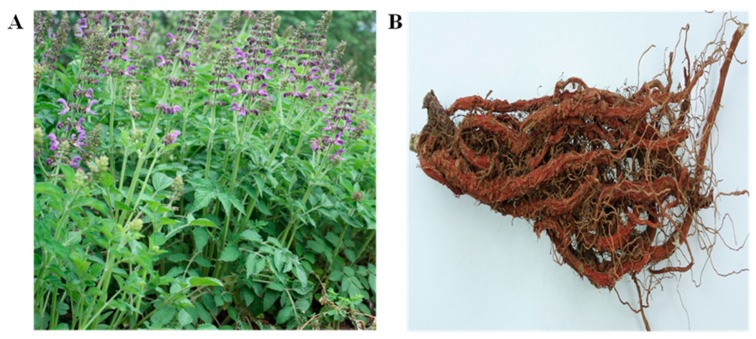
The *Salvia miltiorrhiza* Bunge (**A**) and the raw herb of *S. miltiorrhiza* Bunge (**B**).

Nowadays, traditional Chinese medicines (TCMs) have been recognized as typical representatives of complementary, alternative medicines, which are attracting increasing worldwide attention [1]. Radix *Salviae miltiorrhizae* et Rhizoma (named Danshen in China), the roots and rhizomes of *Salvia miltiorrhiza* Bunge (Figure 1), is one of the well-known TCMs. It has been used for more than a millennium in Asian countries, especially in China, Japan and Korea, and was firstly cited in *Shenlong* *Bencao* *Jing* (200–300 AD, Han Dynasty) [2,3]. The consumption of Danshen in the world exceeds 20 million kilograms per year. In clinical practice, Danshen has been developed into more than thirty pharmaceutical dosage forms for the therapy of cardiovascular disease [4], hepatic injury, pneumonia, chronic nephritis and arthrophlogosis. Among those various preparations, the Fufang Danshen Dripping Pill used for coronary heart disease and angina is the most promising compound Chinese medicine, whose phase III clinical study will be completed in 2016 [5].

Scientific research on the chemical constituents of Danshen started in the first half of the 20th century with the work of Nakao [6] who first discovered and isolated tanshinone IIA from Danshen in 1934. Subsequently, Guo *et al.* reported the existence of protocatechualdehyde in Danshen in 1979 [7]. In the 1980s, thorough and comprehensive research on its chemical constituents was carried out by Luo *et al*. [8]. So far, more than 100 components of Danshen have been isolated and structurally identified [9]. The phenolic acids and the diterpenoid tanshinones plus related quinone derivatives were believed to be responsible for the bioactivities of Danshen all this time. Moreover, scientists and manufacturers have paid more and more attention on polysaccharides from Danshen over the past few years due to their notable pharmacological activities. In the process of practical application, the contents of these components often varied significantly due to different geographic sources [10], harvesting and processing [11], drought stress [12], and thus affected the therapeutic effects of Danshen.

Danshen exhibited various pharmacological activities that were attributed to its chemical constituents. Therefore, comprehensive quality control is critical to ensure the efficacy and safety in clinical use of Danshen. In addition to microscopic and macroscopic authentication, chemical identification, which directly associated with the pharmacological effects, was an important and useful means for the quality control of Danshen. In previous reports, tanshinone IIA and salvianolic acid B were usually chosen as major markers to assess the quality of Danshen and its products [13,14]. However, tanshinone IIA and salvianolic acid B are isolated not only from Danshen, but also from other plants, such as *S. umbratica*, *S. plebeia*, and other *Saivia* species [15,16]. Therefore, for quality control of Danshen, only detecting tanshinone IIA and salvianolic acid B seemed to be partial and insufficient.

The official drug of Danshen was the roots and rhizomes of *S. miltiorrhiza*. The quality of this kind of Danshen was guaranteed and had been proved by clinical application since thousands of years ago. Several other substitute herbs were used in clinical trials, such as *Salvia bowleyana* Dunn, *S. digitaloides* Diels, *S. trijuga* Diels, and *S. plectranthoides* Girff. The presence of these substitutions made the quality control of Danshen more difficult. Due to the lack of effective identification methods, the situation became more and more serious. The methods for analyzing phenolic acids, tanshinones and polysaccharides contained in Danshen as well as their chemical structures, and isolation were reviewed in this paper.

On the basis of that, chromatographic fingerprint analysis was suggested to perform the quality control of Danshen at the beginning of 2000 [17,18]. Chromatographic fingerprint emphasized the systematic characterization of the composition, and identification and evaluation of sample stability. The entire pattern of compounds could be used to determine not only the absence or presence of desired markers, but also the complete set of ratios of all detectable constituents. It was noteworthy that many chemometric methods were combined with fingerprints so as to expand the application of fingerprint to quality control of TCMs [19,20]. The development of the chromatographic fingerprint of Danshen was also discussed in this review.

The chemical components, pharmacological effects and clinical use of Danshen has been generalized in various respects [21,22,23,24,25]. However, the significance of analytical methods in Danshen has just been realized in recent years. Li *et al.* [26] gave a summary on the analysis of Danshen except for its polysaccharides with 108 references in 2009. This review mainly summarized the recent advances in chemical analysis of Danshen and its preparations since 2009. Recently established methods were reviewed, including spectroscopy, thin layer chromatography, gas chromatography, liquid chromatography (LC), liquid chromatography-mass spectrometry (LC-MS), high speed counter current chromatography (HSCCC), capillary electrophoresis, electrochemistry, and bioanalysis, which would effectively advance the establishment of better quality control methods for Danshen. Especially the analysis of polysaccharides in Danshen was summarized for the first time in our review. With the aim of providing constructive and meaningful references for the qualitative and quantitative analysis of Danshen, this review compared and discusses in depth the advantages and disadvantages of these analytical methods in Danshen qualitative and quantitative analysis, which would help for establishing better quality standards of Danshen, and help the readers and especially those newcomers in this area to choose which method was more suitable for their research purposes.

## 2. Chemical Compounds

### 2.1. Phenolic Acids

#### 2.1.1. Chemical Structures

**Figure 2:**
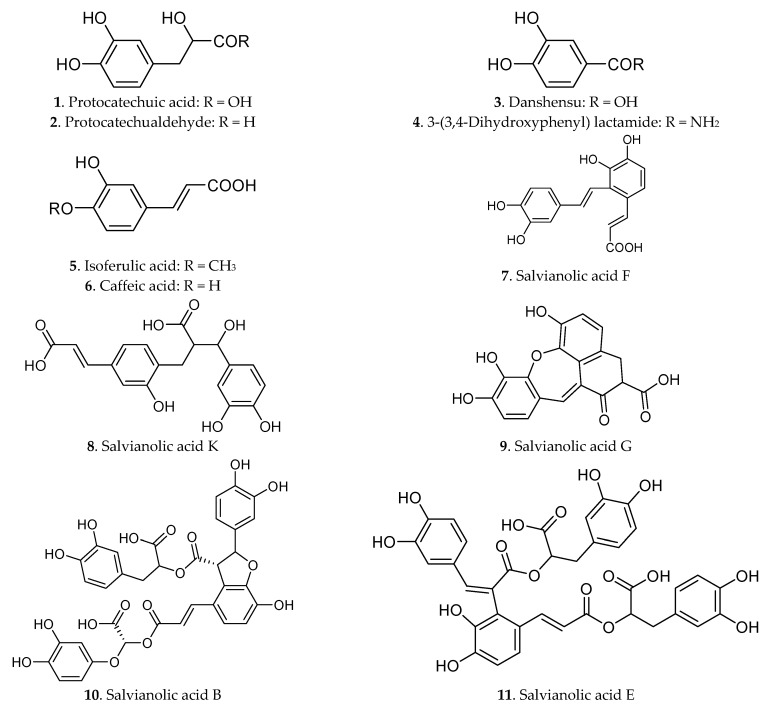

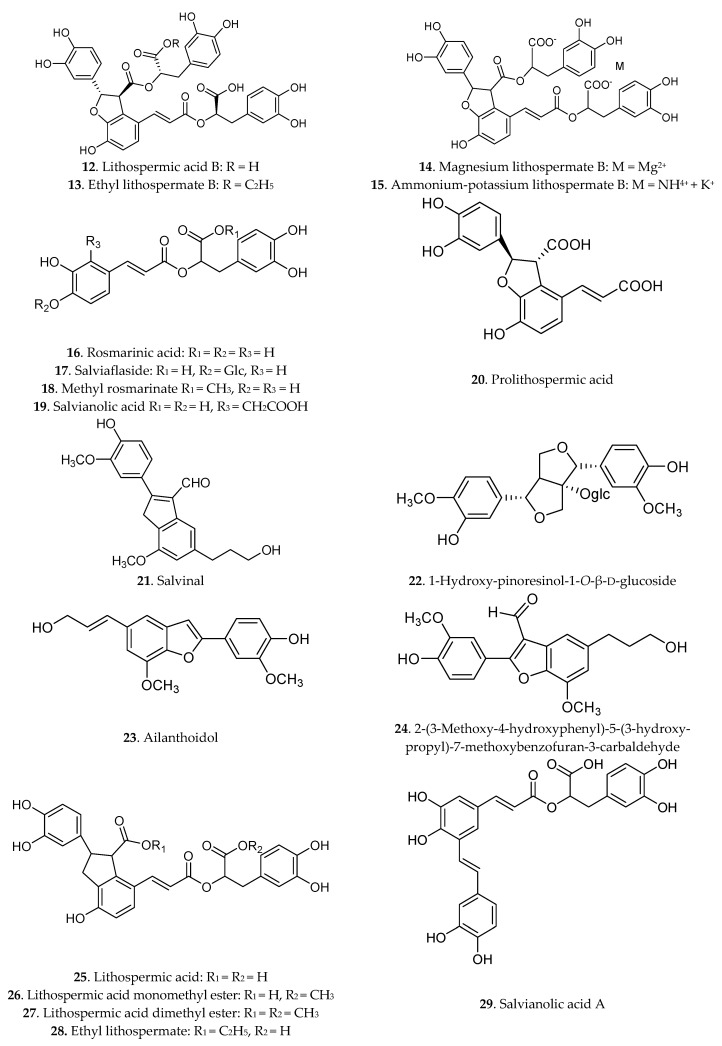

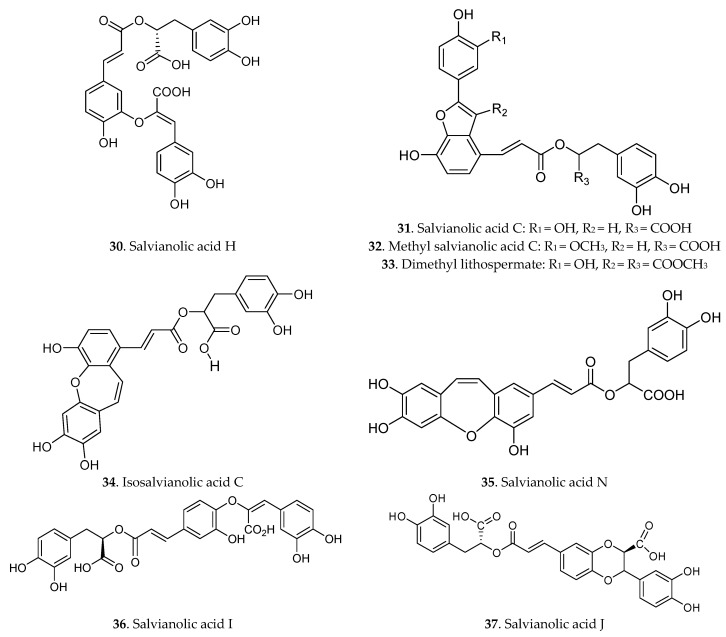
Phenolic acids in Radix *Salviae miltiorrhizae*.

The phenolic acids have attracted the attention of scientists in the last 20 years because of their notable pharmacological activities and the conventional use of herbs by decocting with water. More than 30 phenolic acids (compounds **1**–**37**) had been isolated from Danshen [8,25], as shown in Figure 2, including salvianolic acid A salvianolic acid B, salvianolic acid C, rosmarinic acid, protocatechuic acid, protocatechualdehyde, carnosol or danshensu, lithospermic acid and other derivatives.

Salvianolic acid B, first isolated from Danshen in the 1980s in China, is constituted of caffeic acid dimers [27] and was usually used as one of the markers to assess the quality of Danshen and its products. The majority of the phenolic acids in Danshen were composed of caffeic acid derivatives [28]. Caffeic acid plays a central role in the biochemistry of Danshen and occurs predominantly in the dimer form as rosmarinic acid. Caffeic acid is the building block of a variety of the plant metabolites from the more simple monomers to multiple condensation products with danshensu to give rise to a variety of polymers in Danshen [29].

#### 2.1.2. Biological Activities

With the finding of the cardiovascular-protective effects in the decoction of Danshen abounding in phenolic acids [7], this research verified that the phenolic acids also contributed significantly to the activities of Danshen. In recent years, they have been proved to have significant biological activities including anti-oxidant [30], anti-osteoporosis [31], anti-atherosclerosis [32], anti-diabetic [33], anti-hypertension [34], anti-wrinkle [35], neuroprotective effects [36], and potential therapeutic effect against human immunodeficiency virus [37].

#### 2.1.3. Isolation and Sample Pretreatment

A number of methods had been used to extract phenolic acids from Danshen. These methods mainly included reflux extraction, continuous refluxing at room temperature, and sonication, among which continuous refluxing was the most frequently used. Different solvents such as methanol, aqueous methanol (75%–95%, *v*/*v*) and aqueous ethanol (60%–95%, *v*/*v*), were used for the extraction. Although the conventional methods above were used in most experiments, a majority of them were time-consuming, and might make phenolic acids transform into other compounds.

In recent years, novel methods which are more rapid, economical and efficient have been used for the sample preparation of Danshen. An *in-situ* method by integrated expanded bed adsorption [38] was introduced to extract and separate salvianolic acid B with 88.0% recovery rate. As microwave-assisted extraction (MAE) has the advantages of reducing solvent usage and shorter extraction time, MAE had been adapted for the extraction of Danshen [39]. Supercritical fluid extraction (SFE) was also used for the extraction of phenolic acids where methanol or ethanol was added as a modifier. For instance, when 95% ethanol was added to liquid CO_2_ at a concentration of 5%, *S. miltiorrhiza* extract could be completely extracted from Danshen at 40 MPa and 60 °C within 60 min. [40]. More recently, Nie *et al.* have applied ionic liquid-modified silica to the isolation of five phenolic acids from Danshen [41]. They further found that silica modified with *N*-methylimidazolium hexafluorophosphate had the most excellent adsorption capacity for target compounds. Additionally, some emerging methods were applied to extract phenolic acids, including ultrasonic extraction (UAE) [26], flash extraction [42], modified microfluidic chip [43], tissue-smashing based ultra-rapid extraction [44] and microsphere resin chromatography combined with microbial biotransformation [45].

### 2.2. Terpenoids

#### 2.2.1. Chemical Structures

**Figure 3:**
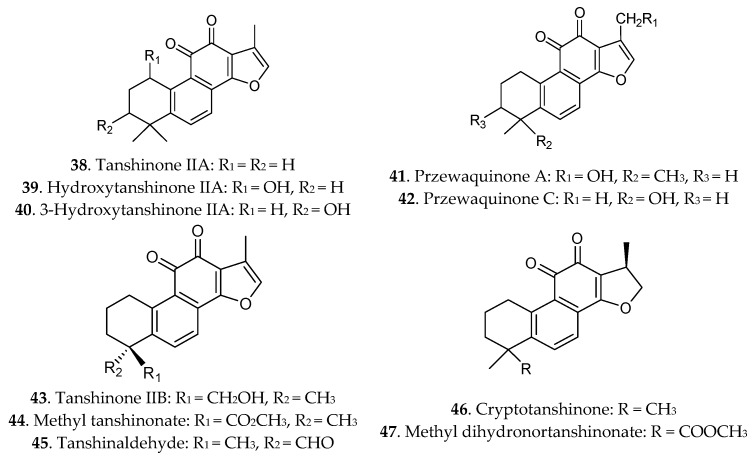

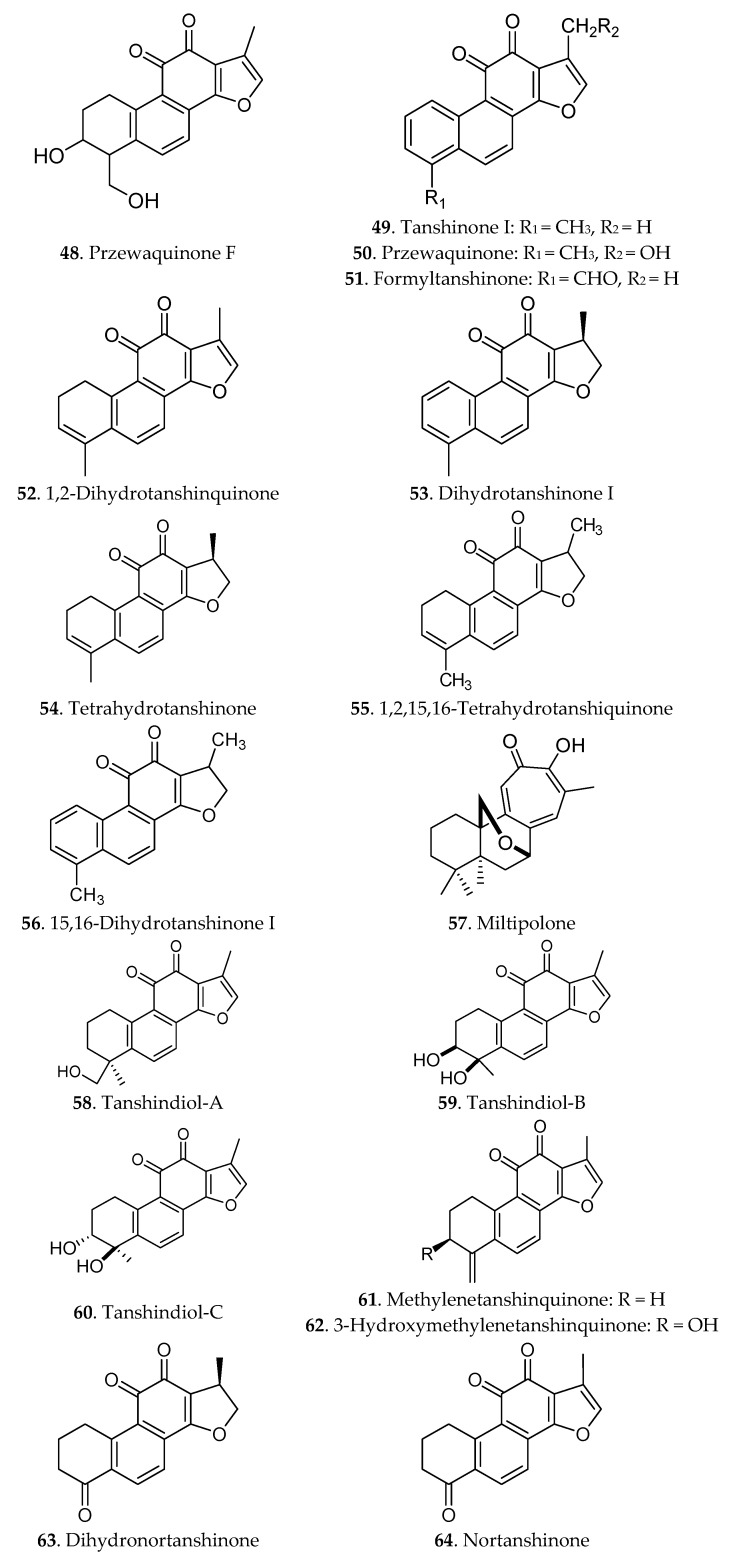

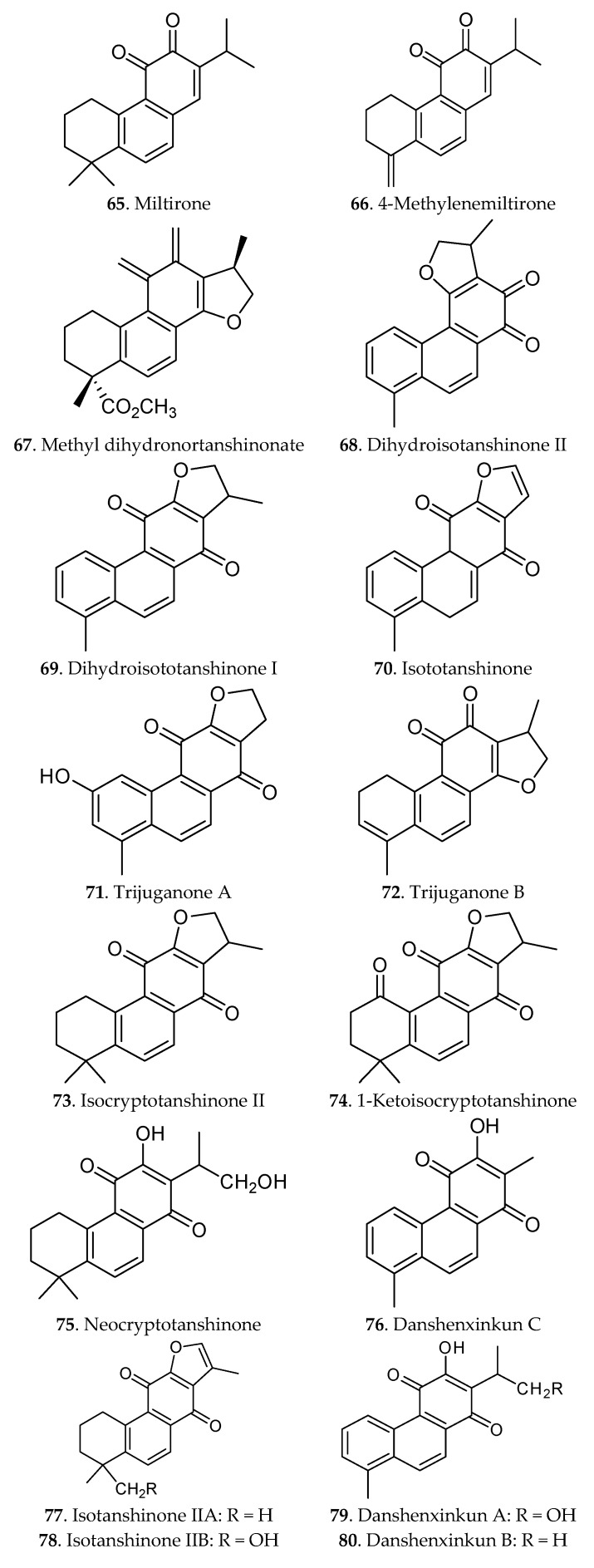

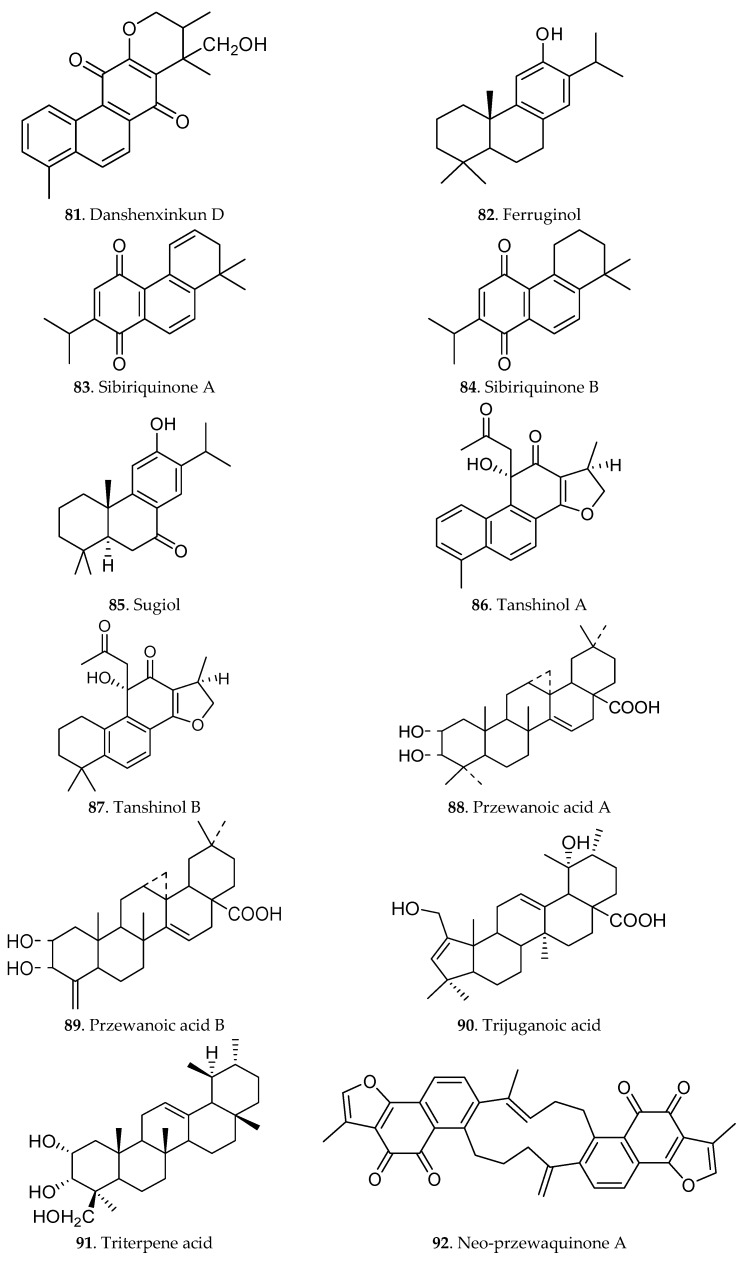
Terpene quinines in Radix *Salviae miltiorrhizae.*

Terpenoids are another effective substance class in Danshen, and they are responsible for the colour of Danshen. So far, at least 40 liposoluble constituents (compounds **38**–**92**) have been isolated from the official species of Danshen. The chemical structures of these diterpenoids [9,46] are given in Figure 3. Most diterpenoids were presented as tanshinones and their analogues containing tanshinone I, tanshinone IIA, cryptotanshinone, dihydrotanshinone, danshenxinkun A, przewaquinone A, tanshinol A, tanshinol B and others. Additionally, some triterpenoids were also isolated from Danshen [47].

The tanshinones are unique chemical constituents in *Salvia* species, and similar compounds are not found in other TCMs [48]. Tashinones are comprised of four rings, including naphthalene or tetrahydronaphthalene rings A and B, *ortho*- or *para*-naphthoquinone or lactone ring C, and a furan or dihydrofuran ring D [49]. Among these, tanshinone IIA and cryptotanshinone, which are characterized by an *ortho*-quinone C-ring, were the most widely researched. Due to the low contents of tanshinones in Danshen, biotechnology strategies were applied to efficiently increase the yield of tanshinones from the cultured hairy roots [50,51].

#### 2.2.2. Biological Activities

These terpenoids exhibited a wide range of pharmacological effects, including anti-tumor [23], anti-microbial [48], antioxidant [52], anti-inflammatory [53], anti-cancer [54], anti-atherogenic [55], anti-diabetes [56], neuroprotective [57], hepatoprotective [58] and immunomodulatory effect [46]. Nowadays, tanshinone IIA has been applied as a potential therapeutic agent for several nervous system diseases [58], including stroke, hypoxic-ischemic brain damage, and Alzheimer’s disease (AD).

#### 2.2.3. Isolation and Sample Pretreatment

Because the majority of the terpenoids were relatively low-polarity, many of the extraction techniques focused on the use of CHCl_3_, ethyl acetate, or petroleum ether as the initial extraction solvent. For more polar compounds, such as tanshinone IIB and przewaquinone, the roots were usually defatted and then extracted with polar solvents such as acetone, *n*-butanol [59] or variable proportions of chloroform-methanol [13]. Reflux, soaking, percolation and ultrasound were the common methods for the extraction of tanshinones. Among these methods, ultrasound was the most effective extraction for tanshinones [26]. Until now, these regular and efficient extraction methods were still widely used with diminutive variations of the solvent type depending on the particular compounds. Though the solvent extraction was time-consuming, it was still the most regular approach because it was inexpensive and simple to conduct. 

However, scientists found that many liposoluble constituents were thermal instability, such as tanshinone IIA, cryptotanshinone and miltirone. The early twentieth century saw the refinement in extraction methods with the use of UAE [60], SFE [61], surfactant-assisted extraction [62], pressurized-liquid extraction [63], infrared-assisted extraction [64] and ionic liquid-based ultrahigh pressure extraction [65] utilized for the extraction of tanshinones. These modern extraction methods had many benefits. For example, SFE, in the absence of co-solvents, was an attractive method for the processing of natural products due to the convenient recycling of the solvent through the adjustment of the temperature or pressure [66]. UAE offered the advantages of an alternative to aqueous surfactant solution, which was an accelerated solvent extraction technique for solid sample matrices, so as to reduce the environmental pollutants [67].

### 2.3. Others

#### 2.3.1. Chemical Compounds

Other compounds were obtained from alcohol extracts, such as β-sitosterol, baicalin, ursolic acid and daucosterol [49,68], while vitamin E and tannin found in certain Danshen extracts and 5,3′-dihydroxy-7,4′-dimethoxy flavanone were isolated from the ethyl acetate extract. In the past few years, the study of polysaccharides from Danshen [69,70] have advanced greatly through comprehensive research.

#### 2.3.2. Biological Activities

Polysaccharides from Danshen shown specific pharmacological effects including cardioprotection [71], immunoregulatory [72], antioxidant [73], antidiabetes [74] and anti-fibrosis [75]. Nowadays, Jiang [76] reported that the hydrophilic polysaccharide extract from Danshen could potentially be effectively developed as an anti-tumor agent with immunomodulatory activity.

#### 2.3.3. Isolation and Sample Pretreatment

Danshen contains different types of compounds in addition to terpenoids and phenolic acids. Reflux, alkali, ultrasonic, and enzyme extraction have been adapted to extract polysaccharides in Danshen [72,73,74]. Among these, ultrasonic extraction could be the optimum method to extract crude polysaccharide. The modified optimal conditions of obtaining crude polysaccharide with ultrasonic extraction were an ultrasonic power of 180 W, an extraction temperature of 54 °C, and an extraction time of 32 min [76]. The crude polysaccharide could be further purified on a Sephadex column by elution using various concentrations of NaCl at different flow rates [74].

## 3. Analytical Methods

### 3.1. Spectroscopy

Several spectroscopic methods, including ultraviolet (UV), infrared (IR) and near-infrared (NIR), were applied in the analysis of Danshen. Different from chromatographic separation, these methods usually exhibited low resolution, and were mainly used to determine the contents of the total terpenoids or phenolic acids, or to establish spectral fingerprints to differentiate Danshen samples from various species and geographical positions.

Comparative evaluation of UV and NIR fingerprint techniques were conducted to control the quality of Danshen based on the common peak ratios and variation peak ratios in the fingerprints [77,78]. According to the differences reflected in SD IR spectra and 2D IR spectra, two types of Danshen of different growth years were discriminated and identified by a three-step infrared spectroscopy method combined with derivative spectroscopy and two-dimensional correlation spectroscopy [79]. The relationship between IR spectra and the chemical compounds was also discovered. Li *et al.* [80] employed FTNIR spectroscopy in transflective mode to accomplish the simultaneous determination of seven phenolic acids from Danshen ranging from 4000 to 10,000 cm^−1^. This method showed similar precision and accuracy to HPLC. Additionally, UV spectroscopy, using multivariate data analysis, was also successfully applied to monitoring the chromatographic process of Danshen extract [81] and the degradation process of several phenolic acids [82]. Recently, a rapid and nondestructive method has been developed for identification of geographical origin and evaluation of phenolic acids of Danshen [83] based on FTNIR spectroscopy combined with discriminant analysis.

The preliminary structure identifications of polysaccharides from Danshen could be also conducted using FTIR. For instance, the purified polysaccharides were ground with KBr powder and then pressed into a pellet for FTIR analysis at frequencies within 4000–400 cm^−1^. After coating with gold, the images of polysaccharides were observed with a SEM system under high vacuum conditions at a 5 KV acceleration voltage, as well as image magnifications of 10,000×, 30,000×, and 50,000× [84]. Generally speaking, the content of saccharides could be measured by the reaction with phenol in the presence of sulfuric acid at 486 nm with glucose as standard; the total content of uronic acid was determined through photometry using *m*-hydroxybiphenyl at 523 nm with glucuronic acid as standard; the protein content of protein-bound polysaccharide was determined using bovine serum albumin as standard.

NMR spectroscopy provides structural information via the number of peaks in the spectrum, their position, spin coupling and the ratio of the heights of integral curves, so NMR technology plays a critical role in the structural characterization and identification of new chemicals from Danshen [47,60,69]. For instance, the degradation products of salvianolic acid A was isolated and identified by NMR and LC-MS [85]. A total of six oxidation products were confirmed, in which four were the degradation products of mono-oxygenation and two were the result of dehydrogenation. Compared with chromatographic techniques, NMR spectroscopy had evident advantage in detecting various metabolites simultaneously, non-destructively and non-targeted [63]. For this reason, NMR was gradually applied to the analysis of a variety of metabolites in Danshen [17,63]. Recently, the method of NMR-based metabolomics in combination with PLS-DA has been developed to detect both primary and secondary metabolites in Danshen and screen potential biomarkers successfully [17]. In the end, Twenty-six primary and secondary metabolites were identified in samples from different regions. The results suggested that this method provided very visualized and effective dissimilarities between Danshen samples.

### 3.2. Thin-Layer Chromatography

Thin-layer chromatography (TLC), a versatile and widely used separation method, was applied to the analysis of Danshen, especially when rapid qualitative and quantitative analyses were required at low cost. The most regularly monitored compound in TLC analysis was tanshinone IIA. The separation of tanshinone IIA was usually carried out on silica gel plates with benzene/ethyl acetate, petroleum ether/ethyl acetate, petroleum ether/ethyl acetate/cyclohexane, *n*-hexane/ethyl acetate/formic acid, *n*-hexane/ethyl acetate, petroleum ether/ethyl acetate/cyclohexane, as the developing solvents. Tanshinone IIA and other terpenoids can be visualized by vanillin/sulfuric acid, ferric chloride/alcohol, sulfuric acid/alcohol, and trichloroantimony/chloroform.

Unfortunately, because of their limited separation capability, poor reproducibility and low sensitivity, TLC methods were usually used to analyze one or a few important compounds in Danshen. Now, improved TLC methodologies have been developed to resolve these problems. Yang *et al.* [86] developed a double-development HPTLC method, which was applied to simultaneous qualitative and quantitative analysis of hydrophilic and lipophilic constituents in Danshen. The separation was performed on a nano-silica gel 60 F_254_ plates using dichloromethane/ethyl acetate/formic acid and petroleum ether/ethyl acetate/cyclohexane as the mobile phases, then their characteristic TLC profiles were observed under UV light at 254 and 365 nm. The simple and precise method indicated that many samples could be analyzed per plate, and could facilitate the quality control of Danshen.

A method of TLC identification using reference extractives [87] was also reported which had a much higher specificity than the single standards, and this method was particularly suitable for a replacement for reference standards that were unstable or difficult to obtain. For the past few years, TLC combined with bioanalysis [88] has been used to screen and isolate active substances in the extracts of Danshen. By using this technique, dihydrotanshinone I, an acetylcholinesterase inhibitor, was isolated from Danshen extract, so this technique could be a valuable tool in the analysis of natural active constituents from complex extracts of Danshen.

### 3.3. Gas Chromatography-Mass Spectrometry

Because of their thermolability, the terpenoids and phenolic acids in *S. miltiorrhiza* could not be analyzed by gas chromatography-mass spectrometry (GC-MS). However, the volatile oil and polysaccharides from Danshen could be detected by GC-MS.

Li *et al.* [89] reported the identification of volatile oil from the leaves of Danshen by GC-MS, which revealed the presence of main volatile components, including hexadecanoic acid, germacrene D, β-caryophyllene, and methyl linolenate. The results indicated that the chemical compositions of the oil from the roots and cultures were quite different from those of the flower oil and leaf oil. Monoterpenes, sesquiterpenes, fatty acids, and alkanes were also identified from the oil of the roots [90].

Recently, some researchers have realized that polysaccharides can be analyzed via GC-MS after acetylation. For example, GC-MS was used to analyze the monosaccharide composition of polysaccharides using a HP-5 fused silica capillary column [17]. According to retention time of the alditol acetate derivatives in GC, Liu *et al.* [72] reported that SMP-W1 was comprised of five different monosaccharides, including rhamnose, mannose, arabinose, galactose and glucose, in the molar ratio of 2.35:2.14:1.27:1.11:0.99. Furthermore, GC-MS might provide a perfect method to analyze the composition of polysaccharides in some TCMs such as Radix *Angelicae Sinensis* and *Astragalus membranaceus*.

### 3.4. Liquid Chromatography

Liquid chromatography (LC) showed many advantages in the chemical analysis of TCMs and their formulations, including high reproducibility and sensitivity, perfect linearity and resolution. So far, LC has been comprehensively used for the qualitative and quantitative analyses of Danshen, and remained to be the major method in the analysis of Danshen. Typical methods were generalized in Table 1.

**Table 1:** LC methods of Danshen and Danshen-containing preparations.

Detection Mode	Analytes	Sample	Stationary Phase	Mobile Phase	Qualitative or Quantitative Analysis	Reference
UHPLC/UV (280 nm)	Multi-components	Danshen herb	Waters Acquity UPLC BEN C18 (100 mm × 2.1 mm, 1.7 μm)	10% 1,4-dioxane and 0.1% formic acid in water–10% 1,4-dioxane and 0.1% formic acid in acetonitrile (0 min: 100:0; 4 min: 80:20; 7 min: 50:50; 14 min: 100:0)	Qualitative analysis	[13]
HPLC/DAD (270 nm)	DSS, PA, Sab, Cry, Tan IIA, Tan I	Compound preparations composed of Danshen and Gegen	Agilent Diamonsil C18 (200 mm × 4.6 mm, 5 μm)	0.1% formic acid–acetonitrile (0 min: 80:20; 3 min: 78:22; 5 min: 70:30; 15 min: 35:65; 25 min: 30:70; 28 min: 30:70)	Quantitative analysis	[14]
HPLC/UV (270 nm)	Dih, Cry, Tan I, Tan IIA, Mil	Danshen herb	Inertsil ODS-SP C18 (250 mm × 4.6 mm, 5μm)	Acetonitrile–0.2% acetic acid aqueous solution (55:45)	Quantitative analysis	[64]
HPLC/UV (288 nm)	Sab	Danshen herb	Agilent Zorbax Eclipse XDB-C8 (150 mm × 4.6 mm, 5 μm)	1% aqueous acetic acid–acetonitrile: methanol (3:2) (0 min: 75:25; 10 min: 65:35; 12 min: 75:25; 17 min: 75:25)	Qualitative analysis	[91]
HPLC/DAD (190 to 400 nm)	Saa, Sab, RA, DSS, PA, Lit	Compound Danshen preparation	Agilent Extend C18 (250 mm × 4.6 mm, 5 μm)	Methanol–acetonitrile–tetrahydrofuran (10:90:0.05) with 50 mM phosphate buffer	Quantitative analysis	[92]
HPLC/DAD (270 nm)	Multi-components	Danshen herb	Agilent Zorbax Extend C18 (250 mm × 4.6 mm, 5 μm)	Acetonitrile–0.02% phosphoric acid (0 min: 61:39; 6 min: 61:39; 18 min: 90:10; 18.5 min: 61:39; 23 min: 61:39)	Quantitative analysis	[93]
HPLC/DAD (245, 262, 272 nm)	Tan I, Tan IIA, Cry	Danshen herb	Agilent Zorbax Eclipse C18 (150 mm × 4.6 mm, 3.5 μm)	Methanol–water (81:19)	Quantitative analysis	[94]
HPLC/DAD (200 to 400 nm)	Saa, Sab, DSS	Danshen herb	Agilent TC-C18 (250 mm × 4.6 mm, 5 μm)	0.05% phosphate–acetonitrile (0 min: 90:10; 15 min: 80:20; 35 min: 75:25; 45 min: 70:30; 55 min: 10:90; 70 min: 10:90)	Quantitative analysis	[95]
HPLC/DAD (200 to 400 nm)	Cry, Dih, Tan I, Tan IIA, DSS, Sab	Danshen herb	Agilent Eclipse XDB-C18 (150 mm × 4.6 mm, 5 μm)	Water–acetonitrile (0 min: 45:55; 16 min: 45:55; 20 min: 20:80; 24 min: 20:80)	Quantitative analysis	[96]
UHPLC/UV (281 nm)	DSS	Danshensu	Waters Acquity UPLC BEH C18 (50 mm × 2.1 mm; 1.7 μm)	Acetonitrile–0.1% formic acid water (0 min: 1:99; 7 min: 1:99: 12 min: 50:50; 14 min: 100:0; 15 min: 1:99)	Qualitative analysis	[97]
UHPLC/DAD (254, 280 nm)	DSS, PA, CA, Dih, Tan I, Cry, Tan IIA	preparation Dan Lou tablet	Aglient Eclipse plus C18 (100 mm × 3.0 mm, 1.8 μm)	0.1 % formic acid–acetonitrile (0 min: 90:10; 1 min: 85:15; 3 min: 78:22; 9 min: 70:30; 11 min: 34:66; 15 min: 34:66; 25 min: 20:80)	Qualitative analysis	[98]
UHPLC/DAD (190 to 400 nm)	Multi-components	Compound Danshen tablets	Agilent Zorbax SB-C18 (100 mm × 4.6 mm, 1.8 μm)	0.1% formic acid–acetonitrile (0 min: 95:5; 7 min: 83:17; 14 min: 75:25; 16 min: 72:28; 17 min: 70:30; 22 min: 45:55; 24 min: 29: 71; 27 min: 15:85; 35 min: 15:86)	Qualitative analysis	[99]
UHPLC/DAD (190 to 400 nm)	Multi-components	Danshen herb	Phenomenex Kinetex HILIC column (50 mm × 4.6mm, 5 μm)	10 mM ammonium acetate–acetonitrile (70:30)	Quantitative analysis	[100]
HPLC/DAD (190 to 400 nm)	Multi-components	Guanxinning injection	Shiseido PC HILIC column (150 mm × 2 mm, 5 μm)	10 mM ammonium formate containing 0.01% formic acid–acetonitrile (0 min: 8:92; 34 min: 28:72; 39 min: 40:60; 42 min: 8:92)	Qualitative analysis	[101]
HPLC/DAD (270 nm)	Cry, Tan I, Tan IIA	Danshen herb	Diamons C18 (4.6 mm × 250 mm, 5 μm)	Water–77% methanol	Quantitative analysis	[102]
HPLC/UV (280 nm)	Cry, DSS, PA, PD, Tan I, Tan IIA, Sal B, Dih	Danshen herb	Waters SunFire RP-18 (150 mm × 3 mm, 3.5 μm)	Water–acetonitrile both with 0.1% formic acid (0 min: 50:50; 30 min: 100:0; 35 min: 100:0)	Quantitative analysis	[103]
HPLC-ED (−0.4 V)	Cry, Tan I, Tan IIA	Danshen herb	Capcell Pak C18 (150 mm × 1.0 mm, 3 μm)	Acetonitrile–water–formic acid (52:48:0.6)	Quantitative analysis	[104]
HPLC/DAD (254, 280 nm)	Sab, Dih, Cry, Tan I, Tan IIA	Danshen herb	Kinetex core-shell C18 (100 × 4.6 mm, 2.6 μm)	0.1% trifluoroacetic acid aqueous–acetonitrile (0 min: 93:7; 10 min: 78:22; 20 min: 67:33; 28 min: 48:52; 31 min: 35:65; 36 min: 19:81)	Quantitative analysis	[105]
HPLC-UV (190 to 400 nm)	DSS, CA, Sab, Sac, RA, PA, Cry, Tan I, Tan IIA	Danshen herb	Merck RP-18e (100 mm × 4.6 mm, 2 μm)	Brij35–cyclohexane–phosphate buffer (pH = 6.60)–*n*-butanol (6.68:0.84:85.56:6.92)	Qualitative analysis	[106]
UHPLC/PDA (280 nm)	Sab, RA, Cry, Tan I, Tan IIA, PA, PD, CA, Fer, I-Fer, Saa, Dih, Prz	Danshen herb	Waters Acquity UPLC BEH C18 (50 mm × 2.1 mm, 1.7 μm)	0.5% Phosphoric acid–acetonitrile (0 min: 95:5; 0.5 min: 90:10; 6 min: 77:23; 7.8 min: 70:30; 9.0 min: 58:42; 11.5 min: 55:45; 14.5 min: 54:46; 17.5 min: 15:85)	Quantitative analysis	[107]
HPLC/DAD (280 nm)	DSS, Sab, PD, Tan IIA, Cry	Danshen herb	Waters SunFire RP-18 (3.0 × 150 mm, 3.5μm)	Water–acetonitrile both with 0.1% formic acid (0 min: 50:50; 30 min: 100:0; 35 min: 100:0)	Qualitative analysis	[108]
HPLC/DAD (280 nm)	DSS, PA, PD, CA, RA, LA, Sab, Saa, Sac, Dih, Cry, Tan I, Tan IIA	Danshen herb	KinetexTM C18 (100 mm × 4.6 mm, 2.6 μm)	0.1% aqueous TFA–acetonitrile (0 min: 93:7; 4 min: 82:18; 15 min: 76:24; 20 min: 67:33; 28 min: 48:52)	Quantitative analysis	[11]
HPLC/UV (280 nm)	Saa, Sab, PD, Cry, Tan I, Tan IIA	Danshen herb	Agilent reversed phase XDB C18 (250 mm × 4.6 mm, 5 μm)	0.1% aqueous phosphoric acid–acetonitrile (0 min: 98:2; 20 min: 83:17; 60 min: 32:28; 70 min: 26:74; 88 min: 70:30; 100 min: 98:2)	Quantitative analysis	[109]
HPLC/DAD (190 to 500 nm)	DSS, PA, RA, Sab, Dih, Tan I, Cry, Tan IIA	Danshen herb	Agilent Zorbax SB-C18 (150 mm × 4.6 mm, 5 μm)	Methanol–water containing 0.5% acetic acid (0 min: 20:80; 10 min: 35:65; 20 min: 40:60; 40 min: 70:30; 50 min: 80:20)	Quantitative analysis	[110]
HPLC/DAD (190 to 500 nm)	Fingerprint analysis	Danshen herb	Waters SunFire C18 (250 mm × 4.6 mm, 5 μm)	Methanol–water (0 min: 60:40; 25 min: 70:30; 35 min: 75:25; 45 min: 90:10; 55 min: 100:0)	Qualitative analysis	[111]
HPLC/DAD (270, 288 nm)	Fingerprint analysis	Danshen herb	Symmetry C18 (250 mm × 4.6 mm, 5 μm)	methanol–water (56:44) for ether extract; 0.5 % formic acid–methanol (0 min: 80:20; 10 min: 60:40; 30 min: 60:40; 45 min: 40:60)	Qualitative analysis	[112]
UHPLC/PDA (280 nm)	Fingerprint analysis	Danshen herb	Waters Acquity UPLC BEH C18 (50 mm × 2.1 mm, 1.7 μm)	0.026% phosphoric acid–acetonitrile (0 min: 85:15; 5 min: 75:25; 8 min: 70:30; 12 min: 50:50; 17 min:10:90)	Qualitative analysis	[113]
HPLC/DAD (200 to 400 nm)	Fingerprint analysis	compound Danshen tablet	Agilent XDB-C18 (150 mm × 4.6 mm, 5 μm)	Methanol–water (73:27)	Qualitative analysis	[114]
HPLC/DAD (190 to 400 nm)	Fingerprint analysis	Danshen Injection	Zorbax Extend C18 (250 mm × 4.6 mm, 5 μm)	0.5% formic acid–acetonitrile (0 min: 90:10; 12 min: 78:22; 30 min: 70:30; 38 min: 55:45; 45 min: 10:90; 50 min: 10:90)	Qualitative analysis	[115]

Note: Saa, (salvianolic acid A); Sab, (salvianolic acid B); Sac, (salvianolic acid C); DSS, (danshensu); RA, (rosmarinic acid); LA, (lithospermic acid); CA, (caffeic acid); PA, (protocatechuic acid); PD, (protocatechualdehyde); Fer, (ferulic acid); I-Fer, (I-ferulic acid); Tan I, (tanshinone I); Tan IIA, (tanshinone IIA); Cry, (cryptotanshinone); Dih, (dihydrotanshinone); Prz, (przewalskin).

HPLC separations were usually carried out on all kinds of reversed-phase C18 (RP C18) or some C8 [91] analytical columns so as to obtain optimized analysis. Due to the acidity of phenolic acids, acidic modifiers were often added to suppress their ionization and to attain the suitable peak shapes and separation. Many modifiers could be selected including formic acid, phosphoric acid, trifluoroacetic acid, acetic acid, or buffer solutions such as phosphate buffer [92,93] or acetate buffer [64]. Ionic liquids, the combination of organic anions and a variety of cations, might also improve the column separation as counterions [94]. In most cases, photodiode array detector (PAD) and UV which were usually set at about 270 nm, and 280 nm, or at other wavelengths for different types of compounds based on their UV absorption were used to monitor the eluate. Moreover, when multiple components differing significantly in chemical structures were chosen as the marker components, the DAD method for multi-wavelength detection that is designed to change during the analysis process was applied so as to get a satisfactory chromatogram [95,96]. In the Chinese Pharmacopoeia, HPLC-UV was the official method for quality control of Danshen [3]. The characteristic compounds, tanshinone IIA and salvianolic acid B were detected on RP C_18_ columns separately. Tanshinone IIA was eluted with methanol/water (75:25, *v*/*v*) and detected at 270 nm. Salvianolic acid B was eluted with methanol/acetonitrile/formic acid/water (30:10:1:59, *v*/*v*/*v*/*v*) at 286 nm. The contents of tanshinone IIA and salvianolic acid B in crude Danshen materials should be no less than 0.2% and 3%, accordingly.

For HPLC, it was difficult to analyze liposoluble and hydrophilic constituents from Danshen simultaneously in a reasonable time, and a gradient elution over 50 min was usually needed. UPLC showed many advantages including reducing run time, increasing power of high resolution, superior signal-to-noise ratio and less solvent consumption. Song *et al.* [13] developed an excellent UPLC method with robust principal component analysis (PCA) to rapidly explore analytical markers for quality control of Danshen using a Waters Acquity UPLC BEH C18 column, whose mobile phase for a binary gradient elution constituted of 1,4-dioxane/formic acid/acetonitrile/water. In addition, UPLC was also applied to detecting chemical compounds from formulated Chinese medicines containing Danshen and the metabolite profiles of Danshen as a result of its high-speed and sensitive features [97,98]. Lv *et al.* developed a UPLC-DAD method for the excellent determination of constituents in Fufang Danshen tablets with little pretreatment of the samples, using an Eclipse plus C18 column and eluting with gradient 0.1% formic acid/acetonitrile with the optimum pH of 7.0–7.7. Finally, more than 40 components from Fufang Danshen tablets were detected by UPLC-DAD within 25 min, as well as 22 metabolites were identified as biomarkers of myocardial ischemia [99].

Aside from having difficulty simultaneously analyzing liposoluble and hydrophilic constituents, some problems still confused researchers when they analyzed Danshen with HPLC-DAD or HPLC-UV. Firstly, conventional reverse-phase columns might not be good for separation of polar compounds, especially for some highly polar phenolic acids found in Danshen. Hydrophilic interaction chromatography (HILIC) columns, which are complementary to reverse-phase columns, have been used over the past few years to work out this problem [100,101]. Lately, a combination of HILIC and RPLC coupled with TOF-MS has been developed for the quality control of Guanxinning injection, a Chinese medicine injection composed of Danshen. Several highly polar constituents including four groups of isomers from Danshen were characterized for the first time by HILIC/TOFMS [101]. The other problem was that HPLC analysis usually lacks adequate reference standards to detect several unknown compounds. To solve the problem of missing standards, Jin *et al.* [102] developed a HPLC method with DAD by the reference extractives for the quality assessment of Danshen. Aside from tanshinone IIA and salvianolic acid B, other terpenoids and phenolic acids had also been selected as marker compounds for the HPLC analysis of Danshen. The determination of other terpenoids, such as cryptotanshinone [64], dihydrotanshinone [103] and tanshinone I [104] have been reported for a long time. Lots of researchers [93,94,103] established a number of validated methods to determine the four tanshinones simultaneously, which suggested that they were the main constituents in tanshinones. Further studies indicated that the four constituents had strong pharmacologic actions [55,56,57].

As for the molecular weight determination of the polysaccharides, this could be analyzed by high performance gel permeation chromatography, using a HPLC equipped with a TSK-gel G4000PWXl column and refractive index detector. Sample solution was injected and run with purified water at 0.6 mL/min as mobile phase, and T-series Dextran was used to establish the standard curve. As a result, its average molecular weight was estimated to be 6.9 × 10^5^ Da [72]. In addition, the composition of neutral monosaccharide could be analyzed on the basis of the following procedure. Firstly, polysaccharides (10 mg/mL) were hydrolyzed in 1 mL of TFA (4 mol/L) at 110 °C in a sealed tube for 2 h. After removing TFA with methanol, the residue was dissolved in NaOH and reacted with 1-phenyl-3-methyl-5-pyrazolone (PMP) to convert the monosaccharides into their PMP derivatives. The PMP derivatives of the standard sugars were analyzed on a Dikma Inertsil ODS-3 column connected to a HPLC system [79].

The majority of mobile phases in HPLC or UPLC analysis of Danshen use large amounts of toxic organic solvents, resulting in a serious environmental impact. By using columns with small particle size or length, a number of methods could reduce the usage or lower the volume of organic solvent needed [96,103,105]. For example, five constituents from Danshen were analyzed on a Kinetex core-shell technology HPLC column (100 × 4.6 mm, 2.6 μm) by a gradient elution using 0.1% trifluoroacetic acid aqueous solution and acetonitrile within 36 min [105]. Compared with conventional HPLC columns, these methods could accomplish fast gradient elution due to the columns with small particle size. Additionally, a simple and environmental-friendly method to analyze liposoluble and hydrophilic components simultaneously has been established by liquid chromatographic using a microemulsion as eluent (MELC) [106]. In comparison with the common HPLC method, the MELC method displayed a higher separating efficiency in a shorter analysis time. More importantly, it did not need gradient elution in order to lower the unstable base line.

Using LC, organ specific localization [11,107], species differentiation [103], variability [109,110] of main components of Danshen could be obtained successfully and easily by qualitative and quantitative analysis on single or multiple constituents. For example, Zhong *et al.* [107] compared the hydrophilic and lipophilic components between *S. miltiorrhiza*, *S. przewalskii* Maxim, and commercial *S. miltiorrhiza* specimens from various regions in China. Hierarchical clustering analysis (HCA) for investigated compounds showed that *S. miltiorrhiza* was significantly different from *S przewalskii*, an adulterant of Danshen. Further research revealed that salvianolic acid B, rosmarinic acid, cryptotanshinone, tanshinones I and tanshinones IIA were optimized as marker compounds for the evaluation, which was beneficial for the quality control of Danshen.

Chemical fingerprinting (CFP) could provide a general picture of the chemical components of TCMs, and was widely applied to their quality control. Some researchers constructed the chromatographic fingerprint of Danshen [111,112,113] by marking common peaks (12–25 peaks), which were generally the chief components, then evaluated the quality of Danshen by comparing the peak areas of common peaks or by similarity evaluation employing professional analytical software. In order to discover the potential markers in the treatment of insomnia, HPLC-DAD was used to establish the fingerprint of the ether extract and water extract of Danshen [112]. The results suggested that the extract of Danshen possess significant sedative-hypnotic activity. However, the accuracy of chemical fingerprints used for quality control might be affected by the instability of the crude drugs and HPLC conditions, especially the pump pressure. To overcome this disadvantage, UPLC combined with chemometric analysis [113] was established to fingerprint Danshen. According to this method, the UPLC fingerprints of components extracted from thirteen batches of Danshen by methanol were analyzed and the stable results as well as accurate quality of the fingerprints were got. Compound Danshen could also be evaluated by fingerprint [114,115]. Chang *et al.* [115] applied the combination use of HPLC fingerprint and potency fingerprint with principal component analysis to quality control of Danshen injection. Finally, the proposed potency fingerprint could provide not only the total antioxidant activity of whole sample but also the antioxidant activity of the individual chromatographic peaks.

### 3.5. Liquid Chromatography-Mass Spectrometry

Liquid chromatography-mass spectrometry (LC-MS) had been applied to the qualitative and quantitative analyses of Danshen without using reference standards [116,117]. The structures of tanshinones usually had a α,β-unsaturated ketone moiety, while phenolic acids were polar compounds with carboxyl or phenol groups in the molecules, thus could be easily ionized in the electrospray ionization (ESI) source. Chen *et al.* [117] analyzed the phenolic acids by reversed-phase HPLC-DAD/(−) ESI-MS^n^, eluting with gradient mobile phase consisting of 0.5% aqueous formic acid and acetonitrile. 38 phenolic compounds were detected in 50 min according to their UV spectra, ESI-MS^n^ and ESI-MS fragmentations. Protocatechuic acid and caffeic acid exhibited abundant [M − H − 44]^−^ or [M − H − 18]^−^ ions corresponding to neutral losses of CO_2_ or H_2_O, whereas rosmarinic acid, salvianolic acid B showed [M − H − 198]^−^ and [M − H − 180]^−^ ions which were consistent with losses of tanshinol and caffeic acid, respectively. However, because of restricted to limited peak capacity and resolving power of one-dimensional (1D) techniques, the chromatographic separation of Danshen was always insufficient in LC-MS, especially for the minor ones. To solve the problem, a comprehensive 2D-LC system coupled with DAD detector and hybrid linear ion trap Orbitrap mass spectrometry was established to separate and identify the phenolic and diterpenoid constituents in Danshen [118]. A number of 328 peaks were finally detected and separated. Among these peaks, 102 peaks were identified or tentatively identified and seven of them were discovered in Danshen for the first time.

LC-MS was also used for the characterization of Danshen constituents from formulated Chinese herbs. Peng et al. [119] developed a LC-LTQ-Orbitrap mass spectrometry method in both positive and negative modes to quickly define the chemical profiles and control the quality of XKS preparations containing five Chinese medicines containing Danshen. More than 51 compounds were detected by LC-MS and 15 active components, such as danshensu and protocatechualdehyde were from Danshen. Chen *et al.* [120] developed an HPLC-Q-TOF-MS/MS and UPLC-QqQ-MS/MS method for the qualitative and quantitative analysis of the major constituents in Shexiang Tongxin dropping pill, and more than 40 components were identified by LC/MS and 13 major components, including tanshinones and phenolic acids from Danshen, were either identified or quatified by comparison of UV spectra, ESI-MS, and retention behaviors with those of reference compounds or literature data. Nowadays, a simple and sensitive UPLC–MS/MS [121] method has been used to determine four constituents in rat plasma after oral administration of Shenxiong glucose injection. Specific MRM pairs of *m/z* 197.0→135.0, 136.9→108.0, 359.1→197.1 was set for the quantitative analysis of danshensu, protocatechuic aldehyde, rosmarinic acid accordingly and *m/z* 449.2→287.1 for the internal standard (luteoloside). All calibration curves o showed excellent linearity (R_2_ > 0.994) with a LLOQ in the concentration range of 20–210 ng/mL in rat plasma, which was more sensitive than the analysis of HPLC-UV.

Generally speaking, due to the fact that salvianolic acids were highly polar constituents, the molecular ions could only be determined by FDMS or FAB-MS. However, the methylated products revealed a diagnostic fragmentation pattern caused by McLafferty rearrangement of β-(3,4-dimethoxyphenyl) lactic ester at *m/z* 222 and (M^+^ − 222), and additionally, the characteristic fragment ions at *m/z* 191, 181, 163, and 151 could also be observed [118,119,120,121,122]. For most of the constituents, the loss of 180 (caffeic acid) or 198 (danshensu) mass units were characteristic for phenolic acids. While tanshinones usually displayed characteristic peaks for [M − CH_3_], [M − CO], [M − H_2_O], [M − COOCH_3_] and so on [118,119,120,121].

### 3.6. Capillary Electrophoresis

Though HPLC remains the main method of choice for the analysis of herbal medicines, an alternative approach could be provided by capillary electrophoresis (CE). Compared to HPLC, CE showed such advantages as online preconcentration, rapid separation, sensitive detection, and minimal consumption of solvent [123].

In the CE analysis of Danshen, phenolic acids continued to be the most frequently monitored compounds [124]. Xu *et al.* [125] developed a CE method with DAD for simultaneous determination of five phenolic acids in Danshen. Under the optimum conditions, two components showed good linearity (R^2^ > 0.9990) in the concentration ranging from 5.0 × 10^−6^ to 1.6 × 10^−4^ g/mL, and the LOD was 3.1 × 10^−5^ and 6.2 × 10^−5^ g/mL for danshensu and salvianolic acid A respectively. More recently, the combination of CE and HPLC-MS [126] has been developed to explore mammalian target of rapamycin (mTOR) inhibitors from Danshen. A bioassay-guided method was performed by CE in order to discover the inhibitors of mTOR. HPLC-MS was applied to trace the specific bioactivator. Finally, salvianolic acid A and C were found to have the bioactivity of mTOR inhibitors.

### 3.7. Electrochemistry

Recently, electrochemical methods have been gradually used in the analysis of TCMs because they are a kind of rapid, simple, sensitive and inexpensive analytical method. These methods do not require any tedious pretreatment, and involve limited pre-separation. Moreover, these techniques could also provide significant information about pharmacological effects. A number of electrochemical methods were reported to evaluate the quality of Danshen. These methods included microwave digestion polarography for determining seven trace elements in Danshen [127], polarographic evaluation of the cardioprotective effect in the presence of salvianolic acid A [128], recovery and purification of rosmarinic acid using lab-scale electrodialysis [129]. The sensitivity of detection increases obviously when oxidant or both surfactant and oxidant are added. Electrochemical and spectroelectrochemical studies of four tanshinones from Danshen [130] indicated that the antioxidant activity could be enhanced with the increasing angle strain in the reduction process.

In addition, salvianolic acid A sodium has been analyzed using an electrochemistry method. Wang *et al.* [131] determined salvianolic acid A sodium in Danshen by cyclic voltammetry with a glassy carbon electrode. A pair of redox of peaks was observed at + 0.15 V after preconcentration for 150 s in Britton-Robinson buffer solution (pH 3.3). Salvianic acid A sodium was linear in the range of 5.45 × 10^−8^ to 1.09 × 10^−5^ M with correlation coefficient over 0.9990 and the LOD of 5.45 × 10^−8^ M. Zhao *et al.* [132] investigated the electrochemical behavior of salvianolic acid A sodium by electrochemical sensor based on reduced graphene oxide and found that salvianolic acid A sodium could generate a sensitive anodic peak at 0.26 V in phosphate buffer solution (pH 5.0). Under optimized conditions, the anodic peak current was linear to the concentration of salvianolic acid A sodium in the range of 8.0 × 10^−8^ to 2.0 × 10^−5^ mol/L.

### 3.8. Bioanalysis

Bioanalysis usually showed higher specificity and lower detection limits, which were approximately 300 times more sensitive than HPLC. More importantly, bioanalysis could avoid time-consuming sample pre-treatment [133,134,135]. A number of molecularly imprinted solid-phase extractions were reported to analyze the constituents from Danshen. Yan *et al.* [133] developed a selective molecularly imprinted solid-phase extraction procedure for separation of tanshinone I, tanshinone IIA, and cryptotanshinone, which could be a feasible tool for determining the contents of tanshinones in Danshen. The practical measuring range of this method was 0.4 mg/L to 500 mg/L (R^2^ = 0.999) with the relative standard deviations less than 4.2%. Hence, this method was used successfully for the quantitative analysis of tanshinones in Danshen.

Because the specific sorbents were characterized by notable recognition properties, the molecularly imprinted polymer has been employed in solid phase extraction (SPE) coupled with HPLC (MISPE-HPLC) for the determination of tanshinone IIA, tanshinone I and cryptotanshinone in fortified serum [134]. In this improved method, the MISPE-HPLC showed high specificity and good recovery for the determination of screening bioactive compounds. The LOD was about 0.3, 0.6 and 0.4 ng/mL for determining tanshinone IIA, tanshinone I and cryptotanshinone, respectively. Therefore, this new way was faster and more sensitive than the analysis of HPLC. Moreover, the contents of three tanshinones in Danshen obtained by MISPE-HPLC agreed well with those of the HPLC analysis.

For the past few years, the approach of molecularly imprinted combined with solid-phase extraction has been successfully applied in the separation of chemical compounds from Danshen. A series of highly specific and sensitive bioanalysis were established [135,136], including molecular imprinting in ionic liquid-modified porous polymer, and magnetic molecularly imprinted polymers, to separate tanshinones in different matrixes, such as food, natural plants, and biological fluids. In addition, other biological analyses like proteomic assay [137], bioinformatics analysis [138], and the method of immobilized G-protein coupled receptors [139] were also reported to screen bioactive compounds in Danshen.

## 4. Discussion

In this review, we have summarized the recent progress in the chemical analysis of Danshen. Terpenes and phenolic acids were regarded as the main bioactive compounds of Danshen. Numerous analytical techniques were applied as the qualitative and quantitative analyses of these components in Danshen and its preparations.

At present, GC, capillary electrophoresis, electrochemistry and bioanalysis were seldom used for Danshen analysis, but TLC and spectroscopy were mainly applied to preliminary screening and routine analysis because of relatively low cost and high resolution. GC was usually used to determine the content of volatile oil or some polysaccharides with the advantage of high speed and separation efficiency. Although poor reproducibility was the main problem of CE, it showed such advantages as online preconcentration, rapid separation, sensitive detection, along with minimal consumption of solvent, and thus might be preferable to the analysis of low content compounds from Danshen. The electrochemical methods do not need tedious pretreatment and pre-separation, so electrochemical methods were particularly suitable for the analysis of elements in Danshen. When high specificity and low detection limits were needed, bioanalysis would be a better way to conduct in the analysis of specific biomarkers from Danshen. 

Among these methods, HPLC was still a preferable method for the quality control of Danshen. However, the lack of reference substances was an unavoidable problem, so chemical fingerprinting might be a better way to ensure the quality of Danshen. Salvianolic acids could degrade into danshensu and caffeic acid under heating conditions. Therefore, the pretreatment of Danshen was bound to affect chemical fingerprinting. It was necessary for chemical fingerprinting to control sample preparation procedures in Danshen. LC-MS, a significant analytical method, was an important method to ensure the efficacy and safety in clinical applications of Chinese medicine preparations. For the general quality control of Danshen, LC-MS was beneficial to the sensitive and comprehensive characterization of the extracts of Danshen even if pure standards were not available. In view of these advantages, it was not only a proper one for the qualitative and quantitative analysis of one or several compounds but also for fingerprinting of Danshen. LC-MS would also be one good choice for the pharmacokinetic studies and *in vivo* metabolism of Danshen. However, due to the expensive instruments of LC-MS, LC-MS could be only limited in the area of researches.

Some suggestions below which might benefit for quality control of Danshen should be considered: (1) it was inadequate to just choose salvianolic acid B and tanshinones IIA as typical components, and rosmarinic acid, cryptotanshinone and tanshinones I should also be appropriate markers for quality control of Danshen due to their potent pharmacological effects as well as relatively high contents in Danshen. (2) Recent researches showed that the polysaccharides from Danshen have various pharmacological effects. However, the analysis of the polysaccharides from Danshen is still confusing which leads to adverse effects in water-solubility preparations of Danshen, so we should take the analysis of the polysaccharides into consideration in the quality control of Danshen. Danshen was usually compatible with other herbs in water for synergistic interaction in clinical use. In future studies, the efficacy of compound Danshen for cardiovascular and cerebrovascular diseases should be further proved by well-designed clinical tests besides the traditional research way. On the other hand, in the conventional use of Danshen decocted by water only a trace of tanshinones could be obtained, so it is urgent to discover a safe and effective solvent suitable in clinical practice to extract Danshen fully in order to reveal the clinical effects as soon as possible.
